# Exploring mental health needs and services among affected population in a cyclone affected area in costal Bangladesh: a qualitative case study

**DOI:** 10.1186/s13033-020-00351-0

**Published:** 2020-03-05

**Authors:** M. Tasdik Hasan, Gourab Adhikary, Sultan Mahmood, Nowshin Papri, Hasan M. Shihab, Rosco Kasujja, Helal Uddin Ahmed, Abul Kalam Azad, Mahbuba Nasreen

**Affiliations:** 1 Department of Psychological Sciences, University of Liverpool, Liverpool, UK; 2 Institute of Disaster Management & Vulnerability Studies, University of Dhaka, Dhaka, Bangladesh; 3 International Centre for Diarrheal Diseases Research, Dhaka, Bangladesh; 4 Combined Family Medicine and Preventive Medicine Program, MedStar Franklin Square Medical Centre and Johns Hopkins Bloomberg School of Public Health, Baltimore, USA; 5 Department of Mental Health & Community Psychology, Makerere University, Kampala, Uganda; 6 National Institute of Mental Health, Dhaka, Bangladesh

**Keywords:** Natural disaster, Mental health, Perceived need for support, Coastal population, Bangladesh, Qualitative findings

## Abstract

**Background:**

Bangladesh, one of the most densely populated countries in the world has been ranked 9th on the Climate Risk Index for 2017: the 10 most affected countries & 7th on the Long-Term Climate Risk Index: the 10 countries most affected from 1998 to 2017. Every year it is afflicted with various climatic disasters including floods, hurricanes and cyclones. Apart from the obvious devastation of lives and property, there is a huge increase in clinical diseases when these disasters occur. Mental health of affected persons after these disasters is a topic that is often neglected by local and national level.

**Methods:**

A qualitative case study was conducted on perceived need on mental health support & availability of such services in a cyclone affected area in rural Bangladesh. Ten (10) key informant interviews (KIIs) with different stakeholders and ten (10) in-depth interviews (IDIs) with affected people were taken.

**Findings:**

We found that cyclones had numerous psychosocial impacts on the population including acute stress disorder, sleep disorder, post-traumatic stress disorders (PTSDs), generalized anxiety disorders, suicidal ideation and depression. The survivors had specific needs for receiving support. Children, elderly and women were perceived to be more vulnerable. The government and NGOs had no specific action plans and initiatives to address these issues and support the mental health of affected population. There was a visible gap in finding effective ways to provide affected people with the required mental health & psycho-social services (MHPSS).

**Conclusion:**

Resilient, responsive and self-sustaining health systems for this vulnerable population are required. Implementation of effective mental health programs and strong mental health policies remain a challenge in Bangladesh where there is a cultural fatalistic acceptance of mental health issues.

## Introduction

Bangladesh is the 9th ranked country worldwide on the Climate Risk Index for 2017: the 10 most affected countries and 7th on the Long-Term Climate Risk Index: the 10 countries most affected from 1998 to 2017 [1]. The coastal zone of Bangladesh consists of 19 districts covering about 20 percent of the country, is geographically vulnerable to adverse ecological processes [2]. It faces the Bay of Bengal and extends inside up to 150 kilometres from the coast, covers more than 30% total cultivatable land thus considered as an area with significant economic interest. This bay provides a perfect niche for breeding of tropical cyclones, facing one or two severe natural disasters every year. Since decades this coastal area had faced many deadly disasters like Sidr in 2007, Aila in 2009, Viyaru in 2013, Komen in 2015, Roanu in 2016 & Mora in 2017 where the economic loss was more than $4.2 billion [3, 4]. Additionally, adverse consequences of climate change include flood, flash floods, hurricanes, heatwaves, heavy rainfall are very common yearly phenomenon around the country [5]. The Government of Bangladesh has already adopted a comprehensive disaster management plan to mitigate the after effect of natural disasters and to improve responses and recovery management at all levels. But health care delivery is not adequately addressed in this plan limiting to physical health only [5]. Also, the coastal health system is not up to the mark and in some hard to reach areas, no active health care delivery centres can be found. Despite the prevalence of malnutrition, hypertension, salinity related disorders, obstetrical complexities and widespread infectious diseases in this region, there is a definite lack of health workers, infrastructure & health care facilities. Apart from the obvious huge increase in clinical diseases when these disasters occur, mental health of affected population are deeply affected due to personal & social loss that is often neglected at local and national level specially in low resource settings like Bangladesh.

In the country, around 20 million people have been suffering from different types of mental illness as per the recent *National Mental Health Survey 2019*–*19*. 16.8% adults are suffering from any form of mental disorder with highest prevalence of depression (6.7%). We do not have any information on the proportion of vulnerable population—especially costal people who experiences various stressors for developing mental disorders [6]. Very few studies in Bangladesh reported this vulnerability and risk of developing mental health disorders such as depression, suicidal tendencies, sleep disorders, post-traumatic stress disorders and so on [7–9]. Globally, WHO estimates about 30–50% of the disaster affected population suffer from diverse psychological distresses due to severe trauma exposure, experiencing injury or death of family members and limited social support [10, 11]. Post-traumatic stress disorder (PTSD), major depressive disorder (MDD), anxiety, phobia, prolonged grief and behavioural problems are common among disaster victims. A recent quantitative study by Mamun et al. estimated that the prevalence of depression among disaster affected costal women was 64.9% & associated risk factors were lower age group, being an income generating member of the family, disaster-related physical injury, and post-disaster work absenteeism [12]. We observed a gap in exploration of qualitative findings on this issue & thus, attempted to understand this further by conducting a qualitative case study in Koyra (a subdistrict), one of the most disaster-prone areas in rural Bangladesh on perceived need for mental health support & availability of such services among affected population.

## Methods

### Study setting

The study was conducted in Koyra Sadar Upazila (Subdistrict) of Khulna district which is one of the most geographically vulnerable areas in Bangladesh. Though it is the 2nd largest Upazila (consists of 7 unions with total area of 1775.41 km^2^) in Bangladesh with a dense population of 861 people per square kilometre, the literacy rate is low especially among females [13]. The main source of standard health services is at the Upazila Health Complex (UHC), which is sparsely staffed. The number of community clinics is twenty (20) and family planning clinics is eight (8) [14]. Any of theses centres have no specific services for mental health care. This study was done in Koyra Sadar Union, central to the 7 unions of the subdistrict.

### Study length

This study was conducted between January to June 2015 inclusive of topic guide development, data collection, transcription, translation, analysis & primary dissemination.

### Study design

This qualitative study followed thematic analysis [15] using an inductive approach. The topic had three dimensions including psychological state of the affected population after the disaster, perceived need for mental health support, and availability of mental health services in the area. This research approach permitted for the exploration of criss-crossing and discordance between different data extracted from divergent sources. For exploring clear meanings within the data, transparency was ensured by careful analysis at their language-independent meanings. Thematic analysis does not require the detailed theoretical and technological knowledge of approaches such as grounded theory and discourse analysis, it can offer a more accessible form of analysis. One of the strengths of applying thematic analysis was its flexibility which identified repeated patterns of meaning within the data set. Analysis was handled under an essentialist epistemology where essentialism was the view that every entity has a set of attributes that are necessary to its identity and function; thus, the findings sought to report the direct experiences or perception of different groups of participants on the particular issue; a method seemingly appropriate to the study aims. For exploring clear meanings within the data, transparency was ensured by careful analysis at their language-independent meanings [15].

### Study participants

We conducted 10 key informant interviews (KIIs) with the following stakeholders- local health care professional (community health care provider-2), government health professional (medical officer-2), NGO workers (2), relief volunteers (2), schoolteacher (1) & local administrative officer (1). These stakeholders were invited via from Koyra Sadar Upazila Parishad. Ten (10) in-depth interviews (IDIs) with the worst affected people (both male-5 & female-5) by natural disaster were conducted. The mean age of the respondents were 48.96 years (n = 20) with a standard deviation of 10.26 years. More than half of the respondents completed high school. Monthly family income varied as because 10 respondents were pro poor affected by cyclone & their economic status could not be explored as their economy mostly depends on agriculture and the income varies time to time. However, the overall socio-economic context was poor to average. Most of the respondents were Muslim (n = 13) & half of the respondents were female (n = 11). We have not limited the study to a specific disaster rather allowed them to talk on any natural disaster they experienced. In most cases they referred to the two most devastating cyclones include the Sidr (2007) & the Aila (2009).

### Data collection technique

The participants were identified via the local authority (Upazila Parishad) & they were selected using purposive sampling. Sampling was continued until new data did not describe a new concept and a new category was not formed. Written informed consent from all participants was obtained prior to interview. By using a topic guide of open-ended questions, semi-structured interviews (KIIs & IDIs) of approximately 90 min in length, were conducted with each participant individually by the primary researcher. Each interview was recorded by digital recorder. Each interview was transcribed, translated and back translated by research team members, and carefully checked by an assigned member of the team.

### Data storage

Data was stored as per the approved study protocol by maintaining standard confidentiality. In addition to the data collection exercise, all involved researchers received intensive training regarding data confidentiality during data collection and storage. All paper files were kept in a secure locked location (IDMVS) [16] and digital data was encrypted. Passwords was shared by principal investigator only and changed periodically. Participant identification numbers were used to label data only and all possible identifiable information were kept separately from this data.

### Data analysis plan

Thematic analysis approach involves a constant moving back and forward between the entire data set, the coded extracts of data and the analysis of the data that are being produced. Analysis was conducted by the principal investigator (primary researcher) using an iterative procedure [15]. Each interview was transcribed, translated and carefully checked by an assigned member of the research team who was a native speaker to ensure meticulousness. Then the initial codes were identified at a semantic level to understand themes by a careful systematic approach. Sorting of codes was done to develop initial themes. Triangulation of the two data sets was used to highlight different experiences and perspectives of each group and dynamic contrast between data [15]. For establishing rigour in the data, triangulation was used and gradually the analysis moved towards saturation [17]. Then the initial themes were reviewed, refined and named. At this point, identified themes were discussed with the interviewers (primary researcher & co-investigators) to ensure that interpretation was appropriate to participants’ experience to further ensure rigour within the data. Thus, the iterative process produced the final themes and interpretations were conceptualised accordingly [15]. For managing data, Atlas.ti software was used.

### Reflexivity

Reflexivity is a core and critical dimension facilitating the production of data that is of a trustworthy standard in qualitative research. At the data collection stage, reflection can be important in understanding why interactions happen, and explain why a participant may have responded in certain ways. It is also important for researchers during this stage to understand and manage the power balance between the participant and researcher. Training and supervision were provided by the senior researcher of the team (MN) to aid in mitigating any impacts of power within the interview. Also, we anticipated, unexpected & painful challenges may arise as the context was sensitive [18–20]. Reflexivity was particularly important in this setting to enable the researcher to understand how the interview was being constructed and adjusted accordingly as per the objectives of the research [19].

### Ethical considerations

Ethical approval was obtained from *Institute of Disaster Management & Vulnerability Studies, University of Dhaka (IDMVS*) [16]. An information sheet was provided to all participants before written informed consent was requested. As mental health disorders are sensitive topics, that might initiate an emotional response, participants’ breakdown was monitored and willingness to continue was checked regularly throughout the session and post interview. Open-ended questions were used to facilitate participant autonomy within the interview, and all participants were debriefed, made informed of confidentiality protocols and additional privacy measures. In transcripts, analysis and all further reports, pseudonyms were used to protect participant identity. Deductive disclosure was another major concern for researchers where the identification of an individual’s identity can be revealed by presentation of unique characteristics even though direct identifiers (e.g. name, addresses) were removed from data. These characterizations were carefully sorted out to protect identity of the participant. The study involves with two diverse groups of participants; researchers were trained in safeguarding protocols adapted to the local context, received structured supervision and instructed to report any risk to participants.

## Results

Qualitative findings of this study generated four major themes such as post-disaster mental health conditions, perceived need for support, vulnerability related to age, gender and availability of mental health services. We have presented the experiences, perception, thoughts and insights from the in-depth interviews (IDIs) of affected population & key informant interviews (KIIs) of health care professionals, NGO workers & volunteers.

### Post-disaster mental health conditions & perceived need for support

#### Findings from IDIs of affected population

Survivors of natural disasters mentioned sadness, frustration, grief & guilt after such traumatic events. Many of them had continued survivor guilt even after many years because when these disasters strike, they attempted to survive and take care of their own safety. Some frightenedly observed the death of their kids & dear ones. These eventually led to survivors’ guilt. One mother said,

*“I lost my only son in front of my own eyes to the increasing tidal waves. Every night I have nightmares till then and I feel frightened, sad and cry for the whole night”.*

One respondent mentioned that someone found another person’s dead son’s body and buried him. This caused the father of the child to have a psychotic breakdown instantly and it continued for long. He was declared as a *pagol* (crazy) by locality eventually. The respondent also mentioned incidences of conflicts & aggressiveness towards each other in such circumstances.

The cyclone warning system was considered as a catalyst for creating anxiety & panic among local uneducated people by two respondents. They described the situation as, Tsunami alert was announced just before the cyclone Aila (2009) and people rushed to the cyclone centres and took shelter in different places, but the Tsunami was not significant enough to affect them. The next day, they overlooked the signal for Aila thinking that nothing will happen but experienced a horrific disaster. This created panic and stress with warning sirens, any hydraulic horns, thunders & lightening or even loudspeakers trigger feelings of anxiety. This continues to happen.

Most of the affected people mentioned about developing a feeling of helplessness and uncertainty about the future. They specifically stated periods of silence & loneliness, grumpy moments, excessive talking, frustration & excessive anger. One mentioned about denial of reality & period of thinking about what they lost with significant anxiety. Two respondents identified themselves with self-harm attempts & suicidal ideations. The researchers tried to understand the duration of sufferings and they described it as acute phases to year-long symptoms to date (almost 10 years in some cases).

#### Findings from the KIIs of health care professionals, NGO workers & volunteers

One local health care professional described a haunting experience. Two weeks after the cyclone Aila, he with a rescue team discovered an empty land with lots of mud surrounded by flooding everywhere with tidal bore. One middle-aged person and his young son sat silently. They did not answer any question just took some foods and water after repeated requesting. He later came to know from the son that this gentleman lost his wife and two of his daughters during the cyclone. They found one dead body of his youngest daughter and did not find the others. After this trauma, he became totally silent and spent all the time at a place where he once had a house.

One NGO worker mentioned that,

*“I have seen many people after the natural disaster, detached from the home and normal life with intense restlessness & very sensitive with the loss.”*

One government health professional described his experience of dealing with many cases with mental issues during and after any disaster including recent flood during his working placement in that area. He also focused on co-morbidities with mental disorders as most of the cases were presented with physical symptoms and his clinical judgement separated the specific findings related to mental health disorders. He indicated that such natural disasters had a profound impact on family relations & psycho-social factors. He experienced many divorces, domestic violence incidences & contemporary suicidal attempts after such disasters where cases were directly or indirectly related to the disaster events or consequences. The respondent observed lack of self-care mostly in families who lost many members during such disasters.

One of the schoolteachers described the experience of a teenage school going girl after Aila:

*“A girl was found in after 5 to 6* *months of the cyclone with repeated fainting and abnormal behaviour. Prior to the flood & devastation, she was completely normal and among the top of her class. After a series of investigations, we explored that she was a victim of sexual assault in the relief camp and she had never told about her experiences before to anyone.”*

The government health professional reported similar cases of sexual assault or harassments during & immediately after disasters with acute stress reaction, chronic depression, withdrawn, reluctant, depersonalized, indifferent & psychotic cases.

He shared a traumatic event related to AILA. He met a father and his 8-year old son who had lost all members of his family. He explained further,

“*An example of depersonalization was seen in a relief line. A father was standing by holding his 8*-*year old son’s hand and there was no hurry in taking relief by him or his son. Both of them were with empty eyes. When we asked them about taking the relief and food items, the father said calmly, “you can give if you want.” We later found out that they lost all their family members during AILA and from then on, they have no interest in living”*

### Mental health vulnerability related to age

Age was raised as a factor that increases one’s susceptibility to developing mental health problems. Children and elderly were pointed out as most vulnerable because they tend to depend on others for shelters, transportation, collecting food and toileting during emergencies. They often remain frightened and panicked longer and need more time than others to come back to the normal life.

Whilst talking about children, a respondent described that children were unable to share their experience and kept the fear inside their mind for a long time. For many, the experience of a natural disaster was very new, and they might not have even heard about such devastation before. One NGO worker explained that, he observed many frightened children who saw many dead bodies, lost homes, schools, playgrounds, lost books, stationaries, toys, pets, stayed in the shelter houses without food, drinking water, and electricity and sometimes without their own parent. He identified changed pattern of conversation, attitudes and playing games among them. He further added:

*“After the cyclone (Aila), I found many children of Koyra were playing grave*-*grave, burial-burial. They made dolls and buried them in clay made burials. Even some of them have made small trees and were destroying them by false sound of wind remembering the disaster.”*

One local volunteer gave an example of a 9 years old boy who was afraid of passing by a pond where he has seen couple of dead bodies including his playmate. This little boy suffered almost for 2 years. A very important observation on vulnerability of adolescents and young people was made by two respondents. The government medical professional identified the complexity of these age groups as they were too young to cope up with the emerging mental health challenges arising from devastations but severely overlooked by adults. The concrete operational stage of cognitive development of a child occurs in this age group (7–11 years), so this sort of experience caused a long-term scar in their cognition that hampered their personality in long run. He also suspected cases of drug addiction might have had relation with this vulnerability which was not investigated properly. Three survivors blamed their poor status as root cause of fragility in such contexts.

Four respondents also mentioned old age as a factor that could make one more susceptible to developing mental health conditions. In addition to the physical vulnerability they suffered psychologically more mostly after the disaster due to loss of carers, financial stress & anxiety induced by loss of income-generating personnel of the family and exposure to devastation. One elderly respondent experienced severe mood disorders after extended rainfall & flooding last year but never expressed as he had no idea that such symptoms have medical remedies. He explained these symptoms persisted long with other occasional episodes of insomnia & anger.

### Mental health vulnerability related to gender

Most of the affected population (8), we interviewed described being female increases susceptibility to developing mental health conditions. One respondent explained, female take care of the young children, cook or manage food, collect water in a challenging environment so they cannot take care of themselves during disasters and afterwards heave workloads with no care makes them vulnerable to develop psychological disorders easily.

One NGO worker gave a different but very pragmatic perspective on this issue saying,

“…*during a disaster age is a vulnerable factor but post*-*disaster gender factor is the most vulnerable”*.

He explained, if a young lady lost her husband during a cyclone (disaster), she cannot live alone or earn to live. Everyone tries to exploit her starting from the relief workers to the sub district members and chairman. It is difficult to protect her from sexual abuse in the society. All these negatively contribute to her mental health condition. One government health official tagged the female as emotionally vulnerable group. He further explained,

“*Female cry openly and become sad easily. They can express their feelings, but men are different in such expressions”.*

He experienced some cases where men are addicted to drugs after devastating disasters (both Sidr & Aila). They had extreme fear of becoming workless which led to drug abuse chronic depression, anxiety & suicidal ideation. He stated,

*“I have seen new cases of drug users just after disaster(s) specially among young male group. They manage these drugs in high prices and often get involved with violence, illegal trades as a short cut measure. Many of them said, they fear no job (joblessness) and no money. I managed suicidal attempts by adult male (earning member of the family) in such circumstances.”*

### Availability of mental health services

We asked about availability of mental health services in the locality and some common statements or concerns were portrayed by the respondents. The medical authority mentioned presence of 27 community clinics and one “fully functioning” health complex with health care services and no psychiatrist. Key informant interview respondents were concerned about the shortage of mental health professionals in the locality to meet the population mental health needs after each disaster. They stated that when disasters strike, institutions think of materialistic help including relief materials, oral saline, emergency physical trauma management kits, but not any support for mental health care. One respondent mentioned that only one psychiatrist give service only in divisional level hospitals and sometimes it’s difficult to reach him because of transport, money or time. Many respondents said they have never seen any doctor providing any service, prescribing drugs or counselling people for mental health issues at the grass roots except for severe mental health disorders. After Aila & Roanu, some programs have addressed mental health issues briefly, but this was usually short term and insufficient.

On a different note, during in-depth interviews, two respondents said that they never thought that such psychological problems have medical treatments and they can seek help for the symptoms. One respondent mentioned that it’s shameful to go to hospital for mental health symptoms as money should not be spent on symptoms without physical pain. One respondent mentioned,

*“…after seeing the most deaths in one day I lost my normal speech and logical thinking capacity, but I have never talked to any doctor about this as I thought they will laugh at me.”*

One NGO worker stated that he noticed presence of complementary medicine services such as homeopathic, unani, ayurvedic treatment provision in every sub-district in national budget but nothing was mentioned about provision of mental health care in grass-root level. He didn’t notice any substantial post-disaster plan by the government to utilize the professional or create such professionals where psychiatrist does not have position in district level hospital.

Key informant respondents mentioned that when people are fighting for basic needs, mental health always comes second line. The medical professional mentioned that,

“*it is not possible to make a dramatic change only by physical help but can be possible by psychological treatment.”*

He emphasized on availability of psychotropic drugs, recruiting mental health nurses and reduction of stigma related to mental health by mass-awareness. Most interviews turned as portrayal of general discussion on mental health status rather than confining to mental health after disaster. Many respondents stressed on specialized mental health support after such disasters as they personally have seen couple of incidences or experienced periods of vulnerability related to mental health by themselves.

One key informant interview respondent stated that mental health support provision after disasters is not a priority for government as the number of such professional is very low. He also mentioned there is a lack of understanding on importance of mental health at policy level and shared a statement by a high official:

*“Our NGO is closely linked to the Ministry of Health. In one of the meetings, a high*-*ranking governmental official said “what is mental health? What is PTSD? What will I do with the crazy people after disaster? He wondered why we are talking about mental health when people cannot get food, have lost their houses, cattle and other resources.*

An NGO respondent mentioned that many NGOs are actively working in Koyra and have specific agendas to address needs after disasters, but he has never seen any initiative related to mental health. He emphasised on appropriate training to make local persons as mental healthcare giver. Another affected person also mentioned that he will definitely visit a professional to get cured from these haunting symptoms even after many years of that disaster (Aila), but he is afraid to find such a professional on regular basis. He stated if these supports can be made available at the nearest community clinic that will be most convenient for him.

## Discussion

This case study described the suffering of disaster affected population in relation to need for support and availability of appropriate mental health services in a rural area of coastal Bangladesh. Symptoms of depression, post-traumatic stress disorder, anxiety, and grief among others were described. These descriptions are usual, and have been highlighted by researchers in similar [21–23]. As the study highlighted symptoms close to PTSD, Choudhury et al. also described a disaster affected population as traumatized after a couple of months after a massive tornado which killed 600 people in Tangail District of Bangladesh in 1996 [7]. Another survey was conducted after two months of the Cyclone Sidr in 2007 and described that 25% of 750 survivors had post-traumatic stress disorder, 18% were found with major depressive disorder, 16% had somatoform disorder, and 15% had a mixed anxiety and depressive disorder [24]. A school-based study in Orissa, India after 1 year of a devastating hurricane also showed similar results of PTSD among adolescent [25]. Also, post-hurricane Katrina demonstrated higher rate of PTSD mostly on low-socioeconomic group [26]. Though we had no focus on risk factors as we were confined in exploring the need for support in relation to psychosocial disorders and availability of services in affected areas, such focuses are important to discuss for designing appropriate intervention or services for this vulnerable population review reported that tropical cyclone has long term behaviour health effect which can debilitate affected people [27]. As our study findings indicated continued impact of major cyclones even after 10 years, we tried to investigate literatures focusing on duration of impact of natural disasters on mental health. Many studies demonstrated a gradual decline over time in the prevalence of disaster-induced mental health disorders though some indicated very long-term consequences [27]. After 5 years of the horrific Tsunami of the Indian Ocean, a population-based survey was conducted in south India on mental health issues which revealed around 78% of the population are still suffering from different psychiatric morbidity and require adequate support to protect their mental health [28].

Our study findings were consistent with other similar studies pointing out female gender [27, 28] and children as most vulnerable to mental health disorders in low resource settings. Another study identified low socio-economic status as one of the two primary risk factors for developing psychological morbidities mentioning gender as the top one [27]. A review shows direct relation with low socio-economic status & post-disaster mental health disorders [29]. Our case study was conducted in a regular disaster affected area with overall low socio-economic state. Other similar disaster-prone or coastal zones are usually low-lying, vulnerable, poor and with extreme low-resources and greater risk of post-disaster stress. Gender also played a vital role to increase the vulnerability of these areas [29, 30]. Thus, our findings are relevant and important for similar geographical localities in the country and other LMICs. Unlike other studies, we stressed on perceived need and available support for mental health disorders, they experienced after the disasters. We also received some fascinating stories from the participants on how they started developing such symptoms, specific needs and how services can be made available. Generalised stigma on mental health were also discussed by some participants. The impact of climate change is further worsening the scenario in these areas every year with repeated flooding, cyclones, heavy rainfall etc. The population of this vulnerable zone deserves appropriate support and services on regular basis.

A recent report [5] argued that the government of Bangladesh, NGOs, civil society members, the health professionals, disaster management professionals designed the disaster management programme with focus on economic relief, assistance and some sort of medicine, physical treatment and recovery aid for the affected people after natural disaster. Mental health support was, by far never their mandate so the severity of mental and psychological trauma and casualties heightened with years. The report stated that, for the first time in the history, teams consisted of Bangladeshi psychiatrists, psychologists, social workers and other support service staffs contributed to estimate the need for psychosocial care and for providing management in 2007 after the devastating cyclone in many coastal areas. But it was a one-time support in these 10–12 years nothing much happened to change the scenario. The existing National Plan for Disaster Management 2010–2015 has no visible space for addressing need of post disaster psychosocial care [5, 21]. More evidences are needed to establish that these constant victims of adverse impact of climate change needs support from the health system and disaster management authority. It’s good to hear that national level mental health care professionals are strongly urging for inclusion of post-disaster psychosocial rehabilitation service within the National Plan for Disaster Management [5]. The current study with its limited range is pointing to an easily accessible, affordable mental health care provided by primary health care professionals & local volunteers in worse-affected areas. A need for comprehensive training on disaster psychiatry should gradually come to light. In addition to capacity building, infrastructure development, administrative reformation is urgently required in this arena.

## Limitations, recommendations & conclusion

We acknowledge that this qualitative case study was conducted in a limited setting with few interviews considering time & resources available for this study. Another limitation was, we haven’t used any vulnerability assessment tool rather analysed qualitative data on thoughts, perceived assessment on vulnerabilities related to age & gender. The topic guide had scopes to get insights on perceived vulnerability from all participants. We also have identified that on this topic only a few reports are available. Thus, the qualitative findings of this case study are important evidences for future researches.

The findings of this case study are describing traumatic experiences of disaster-affected population & relevant insights of health care service providers in a worst affected area of rural Bangladesh. The respondents admitted that the sufferings were prolonged due to unavailability of appropriate services, lack of relevant professionals and overarching stigma related to mental health. As consequence of climate change, every year countries like Bangladesh are facing several natural disasters not limited to cyclone & flooding only. The need for mental health & psychosocial services is increasing as disorders are being evident by some other contemporary studies though no visible initiative can be seen from public of private sector to address mental health services in disaster management and post-disaster health care plan. We recommend a resilient, responsive, gender-sensitive and self-sustaining health systems for this vulnerable population by considering their perceived need and access to the local services. Implementation of effective mental health programs and strong mental health policies remain a challenge in Bangladesh where there is a cultural fatalistic acceptance of mental health issues.

## Data Availability

Data are available from the authors upon reasonable request and with permission of Institute of Disaster Management & Vulnerability Studies (IDMVS), University of Dhaka

## Abbreviations

UHC: Upazila (Sub-district) Health Complex
KII: Key informant interviews
IDI: In-depth interviews
NGO: Non-Government Organisation
PTSD: Post traumatic stress disorder
LMIC: Low-middle income country
IDMVS: Institute of Disaster Management & Vulnerability Studies
